# Supplementary figures and images for: A Rab6 to Rab11 transition is required for dense-core granule and exosome biogenesis in Drosophila secondary cells
Source: PLoS Genet. 2023 Oct 16;19(10):e1010979. doi: 10.1371/journal.pgen.1010979 (PMC10602379; doi:10.1371/journal.pgen.1010979)

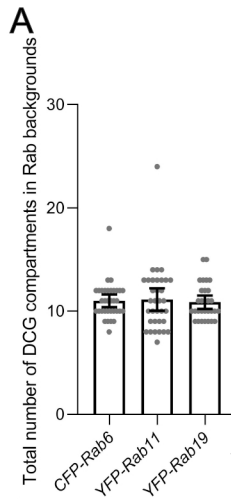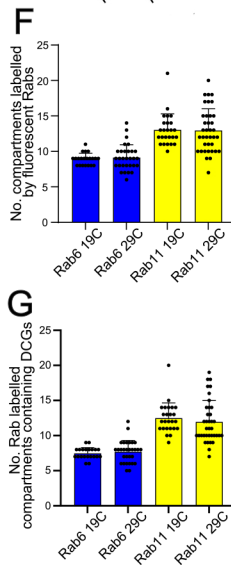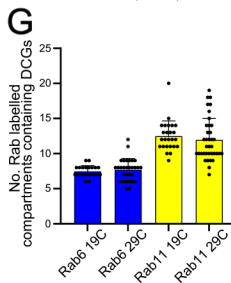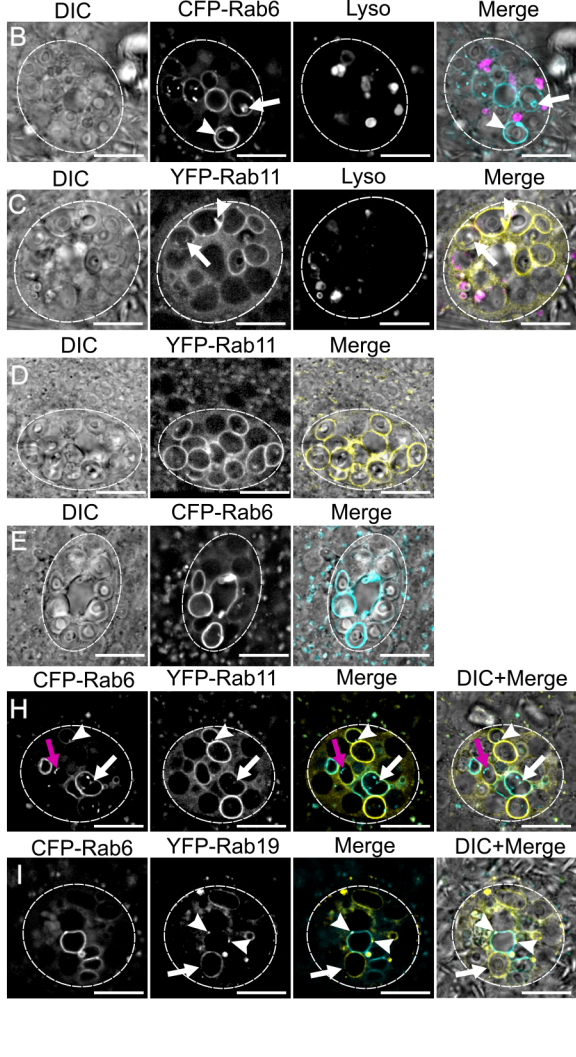

Supplement: S1 Fig — (A) Bar chart showing the number of DCG-containing compartments in control SCs and in SCs expressing different Rab gene-traps, as assessed by DIC microscopy. (B-D) ILVs in SCs associate with DCGs and can form chains extending from the limiting membrane to DCGs. (B) CFP-Rab6-labelled ILVs cluster at the surface of DCGs (arrow) and form bridge-like structures which extend from the limiting membrane to the DCG boundary (arrowhead). (C) YFP-Rab11-labelled ILVs also cluster at the surface of DCGs (arrow) and form bridge-like structures which extend from the limiting membrane to the DCG boundary (arrowhead). (D) SC from YFP-Rab11 male maintained for 12 days at 19°C following eclosion (at 19°C, the accessory gland matures at about half the speed of maturation at 29°C). (E) SC from CFP-Rab6 male maintained for 12 days at 19°C following eclosion. (F, G) Bar charts showing number of Rab6- and Rab11-marked large compartments (F) and DCG compartments (G) in SCs from adults aged at 19°C and 29°C. (H) Clusters of DCG-associated ILVs (white arrow) and ILV chains (arrowhead) can be co-labelled by CFP-Rab6 and YFP-Rab11. Note that there are Rab6-positive puncta inside Rab11-compartments that are Rab6-negative (red arrow). (I) In addition to two or three DCG compartments (eg. arrow), YFP-Rab19 marks microdomains on the surface of CFP-Rab6-labelled compartments, indicated by arrowheads. Data for the bar chart were collected from three SCs per gland derived from 10 glands; bars show mean ± SD. Approximate outlines of SCs are marked by dashed circles. Scale bars: 10 μm. For A, F and G, bars show mean ± SD; CFP-Rab6, n = 34; YFP-Rab11, n = 34; YFP-Rab19, n = 30; Rab6 19C, n = 21; Rab6 29°C, n = 30; Rab11 19°C, n = 25; Rab11 29°C, n = 37. Genotypes for images: (B) w1118; TI{TI}Rab6CFP/+; (C) w1118; TI{TI}Rab11EYFP/+; (D) w1118; P{tub-GAL80ts}, TI{TI}Rab6CFP/P{ryTRiP.HMS02827}; dsx-GAL4/+; (E) w1118; P{tub-GAL80ts}/P{ryTRiP.HMS02827}; dsx-GAL4, TI{TI}Rab11EYFP/+; (H) w1118; TI{TI}Rab6CFP/+ [file pgen.1010979.s001.pdf]

*Rab1-YFP +  $\alpha$ GM130 antibody*

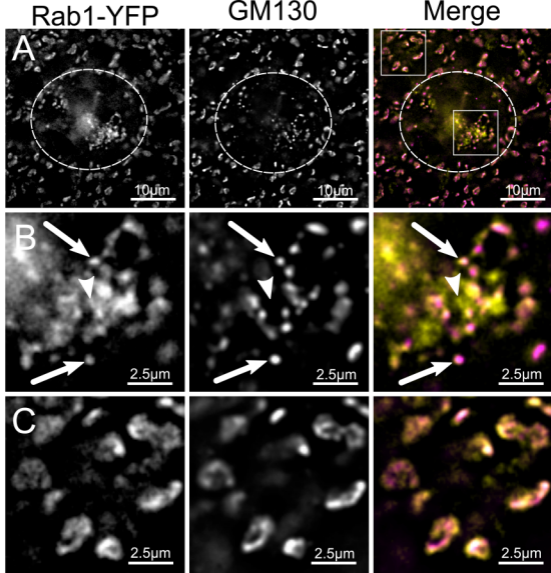

*Rab2-YFP +  $\alpha$ GM130 antibody*

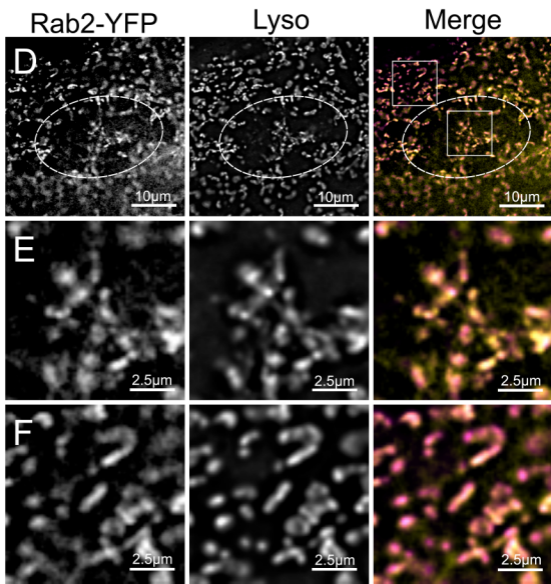

Supplement: S2 Fig — (A-F) Representative images of fixed SCs and surrounding main cells which have been immunolabelled for the cis-Golgi protein GM130 and which express either the YFP-Rab1 (A-C) or YFP-Rab2 (D-F) fusion proteins from the endogenous Rab gene locus. Panels B and E, and C and F represent magnified regions of interest (marked by boxes in A and D) from SCs and the surrounding main cells respectively. Cellular organisation was assessed through wide-field imaging of YFP fluorescence, GM130 immunolabelling, and a merged view of both. (A) Rab1 and GM130 show relatively extensive colocalization within SCs and main cells with most GM130 staining seen in a region close to the centre of the SC. (B) Within SCs, Rab1 and GM130 colocalise on punctate and tubular structures (e.g. white arrows), which presumably represent cis-Golgi compartments. However, Rab1 is also present in many other adjacent compartments not marked with GM130 (e.g. white arrowhead), which likely represent the medial- and trans-Golgi. (C) Within main cells, Rab1 and GM130 also co-localise, but in larger, more tubular structures, and there are some adjacent regions in which only Rab1 is observed. (D) Rab2 and GM130 display extensive colocalization within SCs and main cells. (E) Inside SCs, Rab2 and GM130 share a very similar distribution, both featuring on punctate and tubular compartments near the cell centre. A limited level of adjacent Rab2-only fluorescence is also observed. (F) In main cells, Rab2 and GM130 also strongly co-localise with only a few adjacent compartments labelled by Rab2 only. Genotype for images: (A-C) w1118; TI{TI}Rab1EYFP/+; (D-F) w1118; TI{TI}Rab2EYFP/+. (PDF) [file pgen.1010979.s002.pdf]

Time (minutes)

DIC

YFP-Rab1

CFP-Rab6

All merge

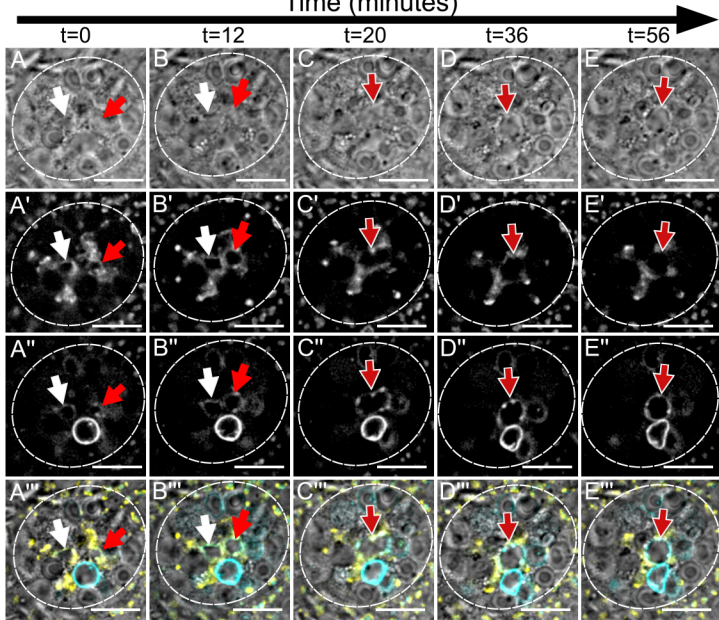

Supplement: S3 Fig — Panel shows ex vivo images of a single SC taken at five discrete timepoints with time since first image shown above in minutes. Rows within panel display cellular organisation at each timepoint through DIC imaging (A-E), fluorescent YFP-Rab1 signal (A’-E’), fluorescent CFP-Rab6 signal (A”-E”), and combined images displaying all three (A”‘-E”‘). Two compartments marked by both YFP-Rab1 and CFP-Rab6 are marked by a white arrow and a red arrow. After the fusion of these compartments, the combined compartment is denoted by a red arrow with a white outline. (A-A”‘) The two central spherical compartments, which are jointly labelled by YFP-Rab1 and CFP-Rab6, initiated their Rab1 to Rab6 transition and expansion in volume approximately 25 minutes prior to time 0 (see S2 Movie). (B-B”‘) After 12 minutes, the two compartments have moved adjacent to each other, as YFP-Rab1 staining gradually diminishes. (C-C”‘) The two compartments fuse to form a single, larger compartment with a distorted shape. (D-D”‘ and E-E”‘) As the time-lapse video continues, the newly formed enlarged compartment regains a spherical shape and loses all YFP-Rab1 labelling. Labelling by CFP-Rab6 continues to increase, resulting in a central, spherical, Rab6-positive compartment which contains no DCG. Approximate outlines of SCs are marked by dashed circles. Scale bars: 10 μm. Genotype for images: w1118; TI{TI}Rab6CFP/+; TI{TI}Rab1EYFP/+. (PDF) [file pgen.1010979.s003.pdf]

CFP-Rab6

YFP-Rab11

Merge

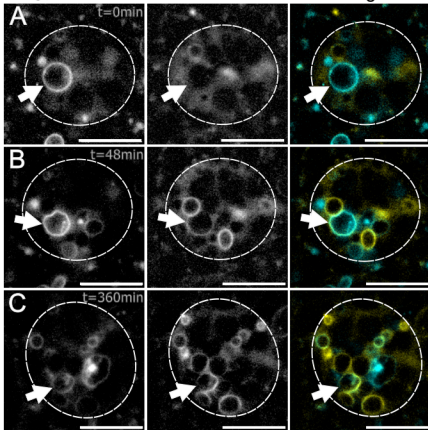

Supplement: S4 Fig — (A-C) Images showing progression of events in the Rab6 to Rab11 transition in SCs expressing the YFP-Rab11 and CFP-Rab6 gene-traps. White arrows highlight a single maturing compartment at three different timepoints during the transition. (A) Prior to Rab11 accumulation, compartments are marked by Rab6 only and are typically spherical with no ILVs present. (B) Following this, Rab11 starts to accumulate on compartment membranes at low levels. Simultaneously, compartments reduce in size and ILV biogenesis (marked by internal CFP-Rab6 puncta in particular) begins. (C) Over the course of many hours, Rab6 is gradually replaced by Rab11 as the primary marker of these secretory compartments; CFP-Rab6 remains visible on ILVs within compartments. Approximate outlines of SCs are marked by dashed circles. Scale bars: 10 μm. Genotype for images: w1118; TI{TI}Rab6CFP/+; TI{TI}Rab11EYFP/+. (PDF) [file pgen.1010979.s004.pdf]

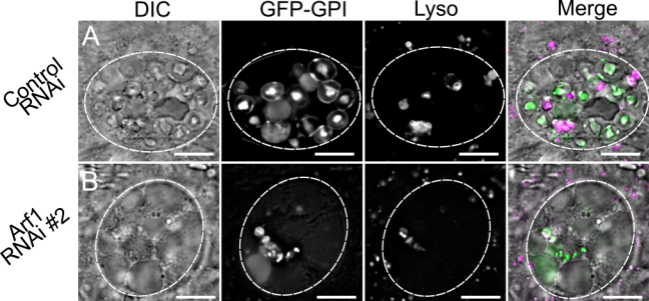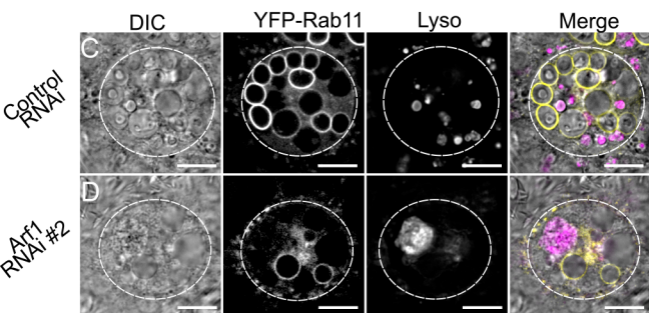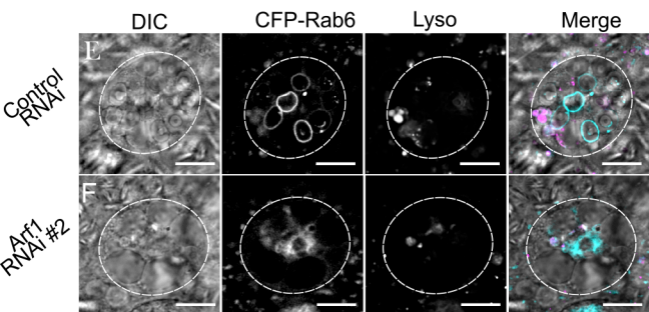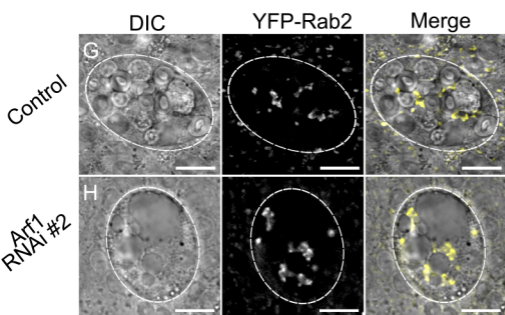

Supplement: S5 Fig — (A-F) Representative images of SCs expressing a control RNAi or Arf1 RNAi #2 in GFP-GPI (A, B), YFP-Rab11 (C, D), and CFP-Rab6 (E, F) backgrounds. (B) SCs expressing Arf1 RNAi #2 contain significantly fewer DCGs than controls (A). (D) Rab11-positive compartment organisation is severely disrupted in SCs expressing Arf1 RNAi #2 versus control (C). (F) Rab6-positive compartment organisation is severely disrupted in SCs expressing Arf1 RNAi #2 versus control. (G, H) Representative images of SCs expressing the YFP-Rab2 gene-trap either alone (G) or alongside Arf1 RNAi #2 (H). When compared to control SCs, the distribution of YFP-Rab2 does not appear to significantly change following Arf1 knockdown and YFP-Rab2 does not mark any large non-acidic compartments. Approximate outlines of SCs are marked by dashed circles. Scale bars: 10 μm. Genotypes for images: (A) w1118; P{tub-GAL80ts}/P{ryTRiP.HMS02827}; dsx-GAL4, P{UAS-GFP.GPI}/+; (B) w1118; P{tub-GAL80ts}/P{Arf1KK101396}; dsx-GAL4, P{UAS-GFP.GPI}/+; (C) w1118; P{tub-GAL80ts}/P{ryTRiP.HMS02827}; dsx-GAL4, TI{TI}Rab11EYFP/+; (D) w1118; P{tub-GAL80ts}/P{Arf1KK101396}; dsx-GAL4, TI{TI}Rab11EYFP/+; (E) w1118; P{tub-GAL80ts}, TI{TI}Rab6CFP/P{ryTRiP.HMS02827}; dsx-GAL4/+; (F) w1118; P{tub-GAL80ts}, TI{TI}Rab6CFP/P{Arf1KK101396}; dsx-GAL4/+; (G) w1118; P{tub-GAL80ts}, TI{TI}Rab2EYFP/P{ryTRiP.HMS02827}; dsx-GAL4/+; (H) w1118; P{tub-GAL80ts}, TI{TI}Rab2EYFP/P{Arf1KK101396}; dsx-GAL4/+. (PDF) [file pgen.1010979.s005.pdf]

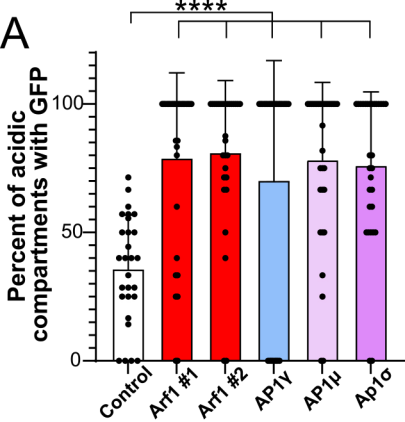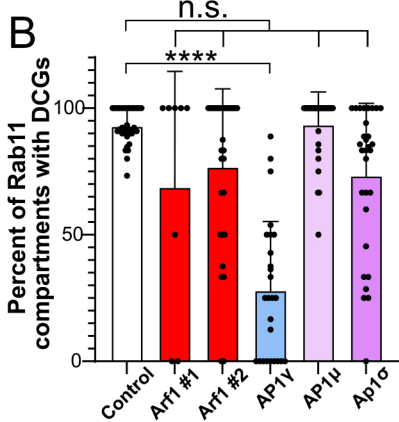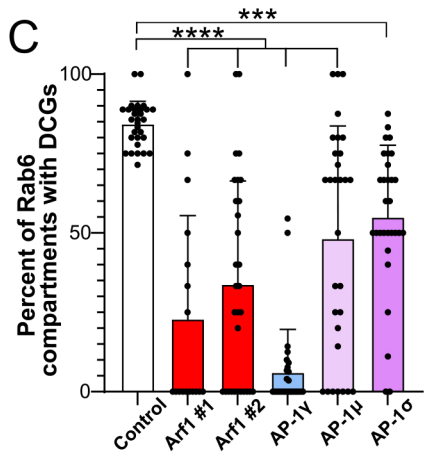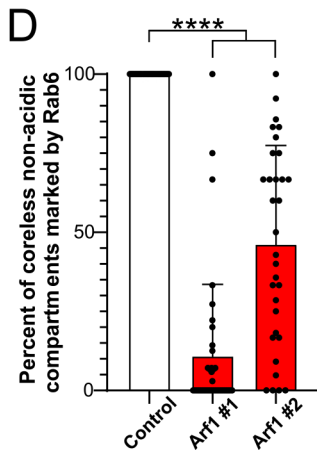

Supplement: S6 Fig — (A) Bar chart showing the proportion of acidic-compartments containing unquenched GFP in control SCs or following knockdown of Arf1 or AP-1 subunits. (B) Bar chart showing the proportion of Rab11-positive compartments which contain DCGs in these different genotypes. Note that despite the significant decrease in DCGs seen in knockdowns (Figs 4F and 5G), the proportion of Rab11-positive compartments which contained DCGs was not significantly affected by any knockdown other than AP-1γ, although most of these DCGs were irregularly shaped (Fig 5H). Therefore, transition to Rab11 identity typically appears to be associated with subsequent DCG formation. (C) Bar chart showing the proportion of Rab6-positive compartments which contain DCGs in these different genotypes. (D) Bar chart showing that knockdown of Arf1 affects the Rab6-positive identity of large non-acidic compartments that do not contain DCGs. For A-D, bars show mean ± SD. For A, Control, n = 28; AP-1γ, n = 28; Arf1 #1, n = 31; Arf1 #2, n = 30; AP-1μ, n = 32; AP-1σ, n = 30. For B, Control, n = 35; Arf1 #1, n = 31; Arf1 #2, n = 36; AP-1γ, n = 30; AP-1μ, n = 34; AP-1σ, n = 30. For C, Control, n = 30; Arf1 #1, n = 39; Arf1 #2, n = 32; AP-1γ, n = 29; AP-1μ, n = 27; AP-1σ, n = 30. For D, Control, n = 30; Arf1 #1, n = 39; Arf1 #2, n = 32. P<0.001: *** P<0.0001: ****. (PDF) [file pgen.1010979.s006.pdf]

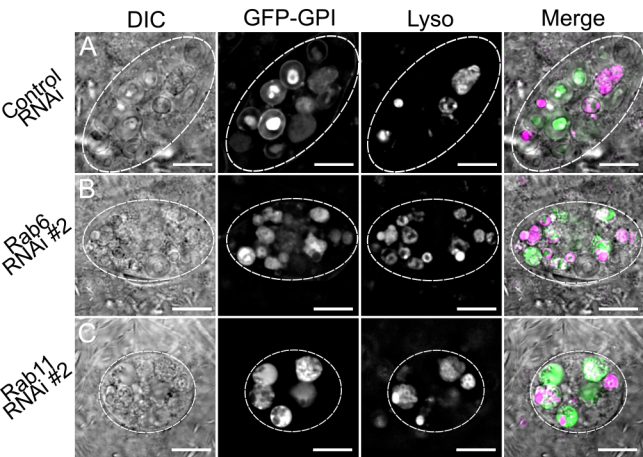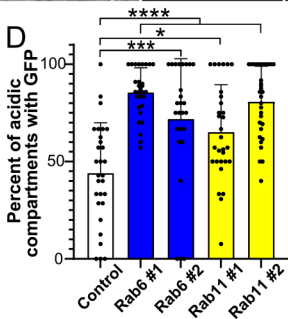

Supplement: S7 Fig — (A-C) Representative images of SCs expressing the DCG marker GFP-GPI with a control RNAi (A) or RNAis (#2) targeting Rab6 (B) or Rab11 (C). Cellular organisation is assessed through DIC imaging, GFP-GPI fluorescence and Lysotracker Red fluorescence, as well as a merged image for each cell. In both the Rab6 and the Rab11 knockdown, significantly fewer DCGs are present and marked by GFP-GPI. (D) Bar chart showing the proportion of acidic compartments containing unquenched GFP in these different genotypes. Approximate outlines of SCs are marked by dashed circles. Scale bars: 10 μm. For D, bars show mean ± SD, Control, n = 29; Rab6 #1, n = 29; Rab6 #2, n = 30; Rab11 #1, n = 30; Rab11 #2, n = 31. P<0.05: * P<0.01: ** P<0.001: *** P<0.0001: ****. Genotypes for images: (A) w1118; P{tub-GAL80ts}/P{ryTRiP.HMS02827}; dsx-GAL4, P{UAS-GFP-GPI}/+; (B) w1118; P{tub-GAL80ts}/+; dsx-GAL4, P{UAS-GFP-GPI}/P{Rab6TRiP.JF02640}; (C) w1118; P{tub-GAL80ts}/P{Rab11KK108297}; dsx-GAL4, P{UAS-GFP-GPI}/+. (PDF) [file pgen.1010979.s007.pdf]

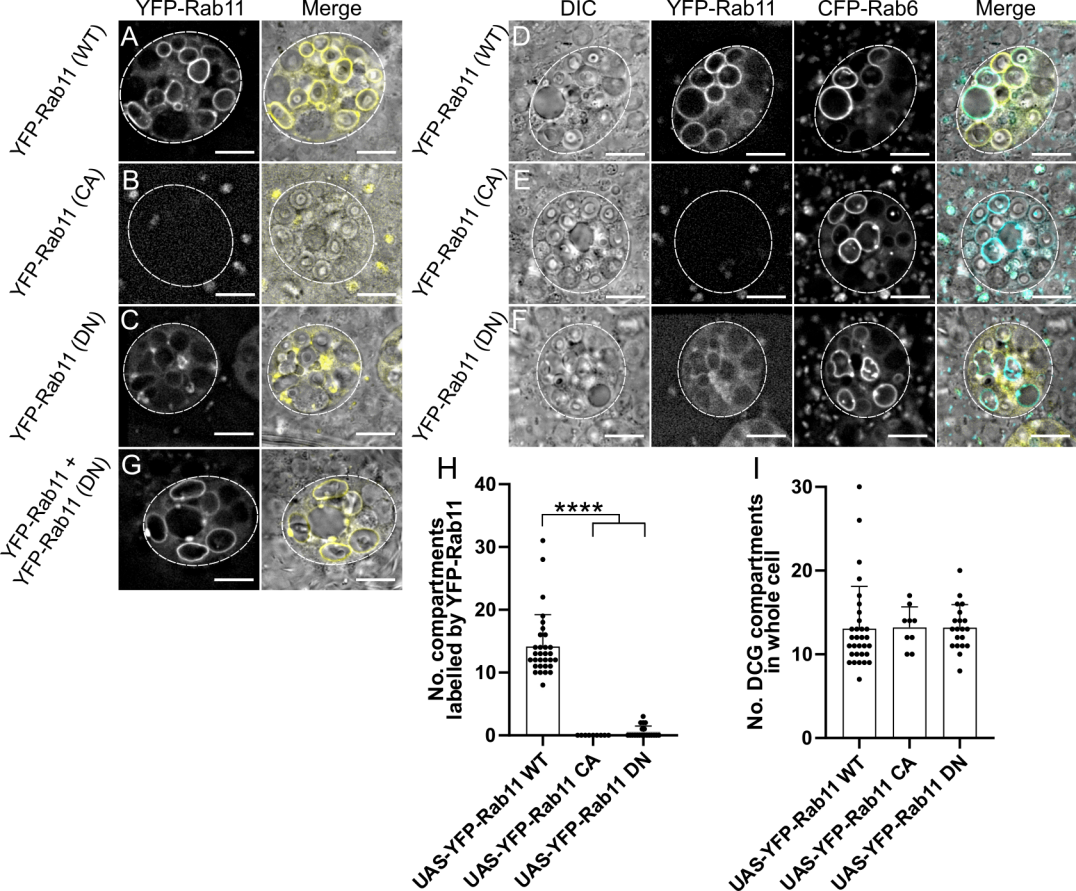

Supplement: S9 Fig — (A-C) Representative images of SCs expressing UAS-YFP-Rab11 fusion constructs that encode wild type (A), constitutively active (B) or dominant-negative (C) Rab11. (D-F) Representative images of SCs expressing UAS-YFP-Rab11 fusion constructs that encode wild type (D), constitutively active (E) or dominant-negative (F) Rab11 in a CFP-Rab6 background. (G) Representative image of an SC expressing the dominant-negative YFP-Rab11 construct together with the YFP-Rab11 fusion produced from the endogenous Rab11 locus. Cellular organisation was assessed through DIC imaging, YFP fluorescence, CFP-Rab6 fluorescence if present, and a merged view of all channels. (A, D) The wild type UAS-driven YFP-Rab11 protein labels all DCG compartments and occasional non-DCG compartments when expressed in SCs, broadly matching the pattern seen with endogenously expressed YFP-Rab11 (Fig 1E). (B, E) The constitutively active YFP-Rab11 construct appears to be only weakly expressed and does not noticeably affect the organisation of SCs. (C, F) The dominant-negative form of YFP-Rab11 does not localise to large non-acidic compartments in SCs, and is instead present at low levels throughout the cytosol and partially concentrated in faintly labelled clusters with a distribution similar to the YFP-Rab1 and YFP-Rab2 fusion proteins. The compartmental organisation of SCs is unaffected. (G) Co-expression of dominant-negative YFP-Rab11 with wild type YFP-Rab11 expressed from the endogenous Rab11 locus reveals labelling of DCG compartments, presumably by the wild type protein. Note the subdomains of concentrated YFP-Rab11 at the outer surface of some of these compartments, which are not observed in controls. (H) Bar chart showing the number of large non-acidic compartments labelled by each YFP-Rab11 fusion protein. (I) Bar chart showing the total number of DCGs per SC following expression of each YFP-Rab11-fusion protein, as assessed by DIC microscopy. For H and I, bars show mean ± SD. UAS-YFP-Rab11-WT, n [file pgen.1010979.s009.pdf]

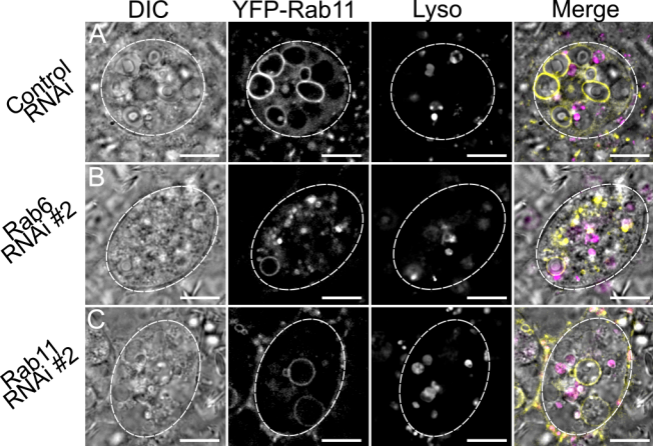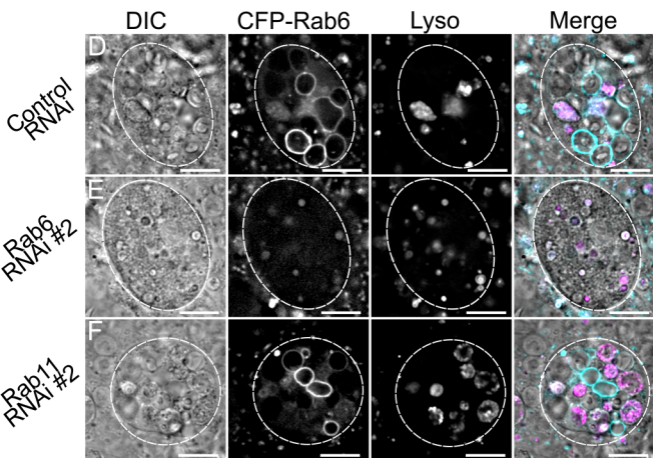

Supplement: S10 Fig — (A-C) Representative images of SCs expressing the YFP-Rab11 fusion gene from the endogenous Rab locus together with a control RNAi (A) or RNAis targeting Rab6 (B) or Rab11 (C). (D-F) Representative images of SCs expressing the CFP-Rab6 fusion gene from the endogenous Rab locus together with a control RNAi (D) or RNAis targeting Rab6 (E) or Rab11 (F). Cellular organisation in all genotypes is assessed through DIC imaging, tagged Rab fluorescence and Lysotracker Red fluorescence, as well as a merged image for each cell. Note that some YFP-Rab11 fluorescence is still visible even after knockdown of Rab11 (C). Approximate outlines of SCs are marked by dashed circles. Scale bars: 10 μm. Genotypes for images: (A) w1118; P{tub-GAL80ts}/P{ryTRiP.HMS02827}; dsx-GAL4, TI{TI}Rab11EYFP/+; (B) w1118; P{tub-GAL80ts}/+; dsx-GAL4, TI{TI}Rab11EYFP/P{Rab6TRiP.JF02640}; (C) w1118; P{tub-GAL80ts}/P{Rab11KK108297}; dsx-GAL4, TI{TI}Rab11EYFP/+; (D) w1118; P{tub-GAL80ts}, TI{TI}Rab6CFP/P{ryTRiP.HMS02827}; dsx-GAL4/+; (E) w1118; P{tub-GAL80ts}, TI{TI}Rab6CFP/+; dsx-GAL4/P{Rab6TRiP.JF02640}; (F) w1118; P{tub-GAL80ts}, TI{TI}Rab6CFP/P{Rab11KK108297}; dsx-GAL4/+. (PDF) [file pgen.1010979.s010.pdf]
