# Supplementary material for: A Rab6 to Rab11 transition is required for dense-core granule and exosome biogenesis in Drosophila secondary cells
Source: PLoS Genet. 2023 Oct 16;19(10):e1010979. doi: 10.1371/journal.pgen.1010979 (PMC10602379; doi:10.1371/journal.pgen.1010979)
Supplement: S8 Fig — (A-C) Representative images of SCs expressing UAS-YFP-Rab6 constructs encoding wild type (A), constitutively active (B) or dominant-negative (C) Rab6. (D-G) Representative images of SCs expressing UAS-YFP-Rab6 constructs encoding wildtype (D), constitutively active (E) or dominant-negative (F, G) Rab6 in a CFP-Rab6 background. Cellular organisation was assessed through DIC imaging, YFP-Rab6 fluorescence, CFP-Rab6 fluorescence (if present), and a merged view of all channels. (A and D) Wild type YFP-Rab6 has a similar distribution to CFP-Rab6 expressed from the endogenous Rab6 gene locus (Fig 1C and 1D). (B and E) A constitutively active form of YFP-Rab6 shows a similar distribution pattern to wild type YFP-Rab6, but labels less secretory compartments. (C) Expressing a dominant-negative form of YFP-Rab6 produces SCs with many small compartments that do not contain DCGs. The limiting membrane of these compartments do not appear to be labelled by YFP-Rab6, some of which is abnormally sequestered into the two SC nuclei. (F, G) When the dominant-negative form of YFP-Rab6 is expressed in the CFP-Rab6 background cells, only a minority of SCs contain many small non-DCG compartments, which are not labelled by either fluorescent Rab (G). The majority of SCs in this genetic background instead produce a mix of non-DCG and DCG compartments that are mostly co-labelled with CFP-Rab6 and dominant-negative YFP-Rab6. A few compartments are labelled exclusively by one or other fluorescent marker (see the white and magenta arrows in F). (H-M) Bar charts showing the number of large secretory compartments, DCG compartments labelled by each YFP-Rab6 construct and total DCG compartments in each cell. (H-J) Compartments were counted in the absence of the endogenously labelled CFP-Rab6 protein, except for the final condition (YFP-Rab6-DN and CFP-Rab6), which leads to a highly variable phenotype. The bar charts show numbers of YFP-Rab6-labelled compartments (H), number of YFP-Rab6-labelled DCG [file pgen.1010979.s008.pdf]

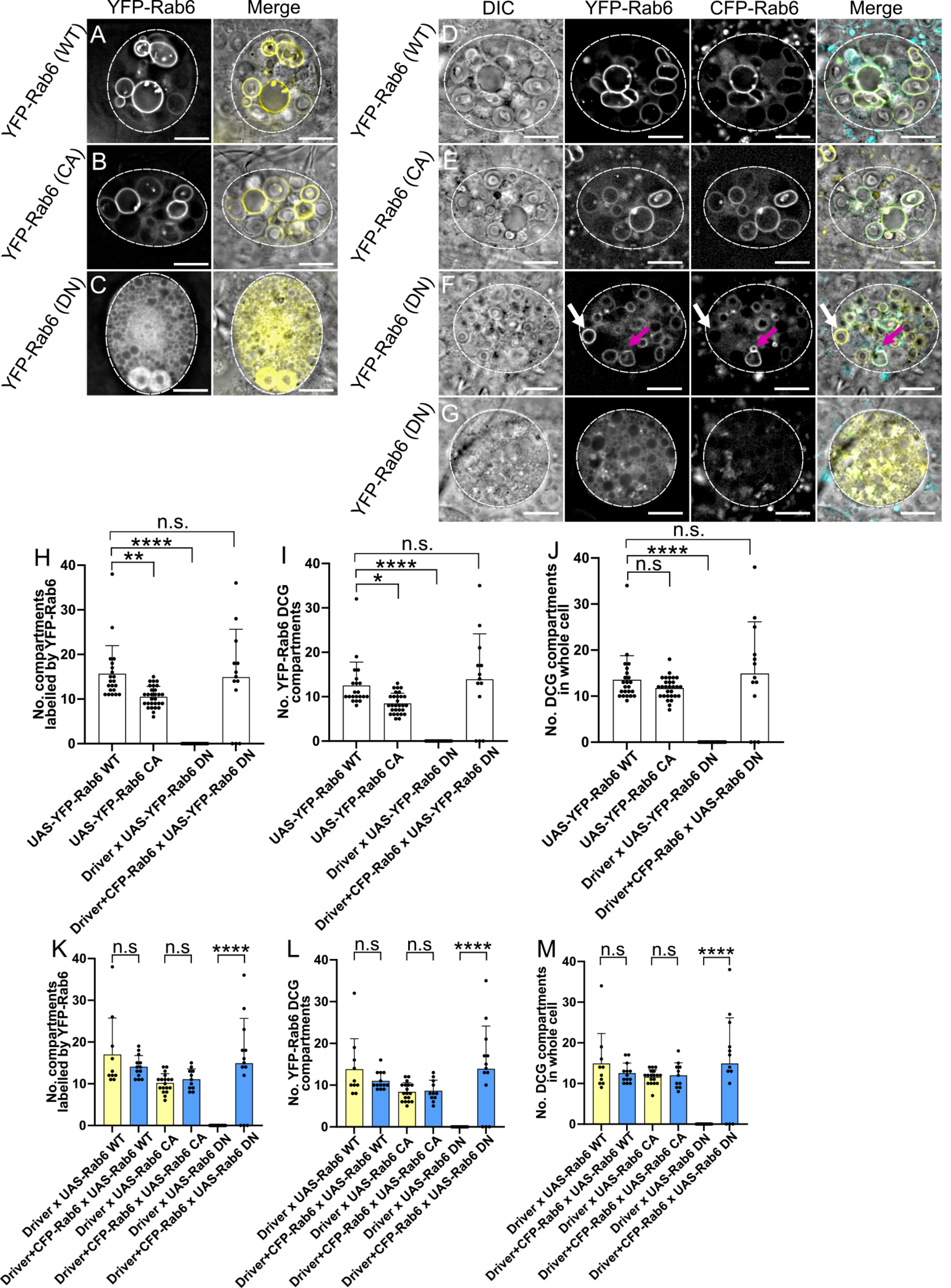
YFP-Rab6
Merge
DIC
CFP-Rab6
YFP-Rab6 (WT)
YFP-Rab6 (CA)
YFP-Rab6 (DN)
A
B
C
D
E
F
G
H
I
J
K
L
M
n.s.
n.s
**
*
****
No. compartments labelled by YFP-Rab6
No. YFP-Rab6 DCG compartments
No. DCG compartments in whole cell
UAS-YFP-Rab6 WT
UAS-YFP-Rab6 CA
Driver x UAS-YFP-Rab6 DN
Driver+CFP-Rab6 x UAS-YFP-Rab6 DN
Driver+CFP-Rab6 x UAS-Rab6 DN
Driver x UAS-Rab6 WT
Driver+CFP-Rab6 x UAS-Rab6 WT
Driver x UAS-Rab6 CA
Driver+CFP-Rab6 x UAS-Rab6 CA
Driver x UAS-Rab6 DN
