# Supplementary material for: A Rab6 to Rab11 transition is required for dense-core granule and exosome biogenesis in Drosophila secondary cells
Source: PLoS Genet. 2023 Oct 16;19(10):e1010979. doi: 10.1371/journal.pgen.1010979 (PMC10602379; doi:10.1371/journal.pgen.1010979)
Supplement: Attachment — Submitted filename: comments on manuscript PGENETICS-D-23-00462.pdf [file pgen.1010979.s016.pdf]

The manuscript by Adam Wells and colleagues provides a step up in knowledge concerning the roles of Rab1, Rab6 and Rab11 in DCG formation in the secondary cells of the Drosophila accessory gland. The most significant advancement relates to the development of a live imaging technique that allowed the visualization of these cells and the follow-up of vesicle compartments and their association with endogenously-tagged Rab proteins during biogenesis and maturation. However, the Rabs transitions, specifically the Rab6 to Rab11 transition that is claimed in the title of the manuscript as required for DCG formation and that would represent the novelty and most significant contribution of the work is not directly tackled. It is important to mention that the presence of these three Rabs on the membrane of these vesicular compartments had been previously reported, as well as its requirement in the development of DCG.

They began by performing a detailed characterization of the subcellular distribution of three different Rab proteins (Rab11, Rab6 and Rab19) in secondary cells of unfixed accessory glands, specifically in relation to large non acidic vesicles, that may or may not contain dense core granules (DCG). These characterizations had been previously reported to some extent using the same endogenously tagged Rab lines but on fixed tissues. They also describe the presence of Rab6- Rab11-or Rab19-labelled internal puncta, likely to be ILV, in the boundaries of the DCG or even in non-DCG vesicles. Live imaging allowed the authors to identify some Rab1-positive compartment that grow and turn into Rab6-positive large compartment that lacks a DCG. Later on, this same vesicle develops a DCG. They identify that homotypic fusion might be the driving force behind the growth of Rab1/ Rab6 compartment. These are descriptions of the live imaging observations and **no mechanistic regulation of the process is provided**.

Also, they detect that these Rab6-positive DCG-negative compartments continue its maturation process by: incorporation of Rab11; internalization of Rab6 and ILV formation, resulting in a compartment with Rab11 externally, Rab6 in ILV and a DCG.

Does the Rab6-Rab11 transition require inactivation of Rab6 or its dissociation from the maturing DCG-containing compartment? Is all Rab6 incorporated into ILV? What happens if Rab6CA is expressed?

Then they analyze the requirement of Arf1 and components of the Ap-1 complex in this process, regulators previously characterized in mammalian cells as important for DCG formation. Important to say is that these proteins are known regulators secretory vesicle emergence from TGN. Therefore, they are expected to be required in general for DCG compartment biogenesis and specifically for DCG

formation. Therefore, in this reviewer opinion this particular point adds on to previously described and extensively characterized role of Arf1 and AP-1 on TGN-derived vesicle biogenesis. Similarly, the reduction in Rab11- and Rab6-labelled compartments upon Arf1 and AP-1 downregulation is expected since no Golgi-derived vesicles are generated when the function of these crucial factors is compromised. Supporting this is the observation made by the authors upon expression of the same RNAis in a genetic background that expresses Rab1-YFP. They detect that Rab1 compartment is enlarged consistent with cargo accumulation at TGN upon Arf1 and AP1 silencing. Again, these are not novel functions for these proteins/complexes. In this point the authors draw a conclusion with which this reviewer disagrees: "The existence of these large compartments marked by Rab1 also suggests that Rab6 is not required for the fusion events between compartments formed in the trans-Golgi and compartment expansion to occur". The authors interpret that Rab1-positive large compartments are a consequence of Rab6-independent homotypic fusion whereas this reviewer tends to think that they are an enlarged Golgi complex that results from accumulation of material that is unable to emanate inside Golgi-derived vesicles. **The authors should provide clear evidence to distinguish between these two possibilities**.

To support the idea that the Rab6 to Rab11 transition is required for DCG biogenesis, the authors should perform experiments to test this more specifically, and not by affecting TGN-derived vesicle biogenesis all together.

Also, the authors try to define temporal requirements for Arf1 and AP-1 complex depending on the severity of the phenotypes obtained upon their knock-downs. It is advisable to make such comparisons based complete loss of function analysis since the different phenotypes obtained could be dew to different degrees of mRNA interference and not real functional differences. **Additionally, the authors could perform a phenotypic characterization at different levels of RNAi expression (by rising flies at temperatures below 29C).**

Later on the authors test weather Rab6 and /or Rab11 are required for DCG biogenesis, and find that both of the do. However the effect is not complete, with some DCG still forming. These could be due to remnant levels of wither Rab6 or Rab11 proteins, supported by the fact that Rab6-YFP and Rab11-YFP are still detectable under silencing conditions of the respective mRNAis. Or, more interestingly it could be due to the existence of more than one pathway controlling DCG biogenesis. **These two possibilities should be evaluated. The effect of Rabs dominant negative isoforms could be tested**.

Rab6 to Rab11 transition taking place during DCG biogenesis is visualized by live imaging. However, its functional relevance is not directly tested. In the first paragraph of the last section of the Results, the authors state that “Where both Rabs were found to be necessary for a process to occur, we concluded that the Rab6 to Rab11 transition was required to facilitate that process.” To this reviewer this reasoning is not adequate and the requirement of the **Rab transition** itself should be tested more directly. For example: can Rab11 associate to de vesicle if Rab6 is not removed from it? Is Rab6 GTP hydrolysis required for the transition? Is Rab6 removal form the membrane of the vesicle dependent on Rab11? Does expression of a constitutively active form of Rab11 accelerate Rab6-compartment maturation, DCG and ILV formation?
