# Supplementary material for: A Rab6 to Rab11 transition is required for dense-core granule and exosome biogenesis in Drosophila secondary cells
Source: PLoS Genet. 2023 Oct 16;19(10):e1010979. doi: 10.1371/journal.pgen.1010979 (PMC10602379; doi:10.1371/journal.pgen.1010979)
Supplement: Attachment — Submitted filename: Response to reviewer comments.docx [file pgen.1010979.s017.docx]

The reviewers appreciated the attention to an important problem, but raised some substantial concerns about the current manuscript. Based on the reviews, we will not be able to accept this version of the manuscript, but we would be willing to review a much-revised version. We cannot, of course, promise publication at that time.

***Our response:*** *We are grateful to the reviewers and editors for their careful consideration of our manuscript and helpful comments. We agree that the focus of our work addresses an important problem, and believe that some aspects of this problem are not straightforward to analyse in other systems.*

Should you decide to revise the manuscript for further consideration here, your revisions should address the specific points made by each reviewer. We will also require a detailed list of your responses to the review comments and a description of the changes you have made in the manuscript.

***Our response:*** *We have addressed all the reviewers’ comments in the revised manuscript and in this document.*

Please be aware that our [data availability policy](http://journals.plos.org/plosgenetics/s/data-availability) requires that all numerical data underlying graphs or summary statistics are included with the submission, and you will need to provide this upon resubmission if not already present.

***Our response:*** *We have included an Excel file containing all the datasets used in the manuscript (SI Data Files).*

While revising your submission, please upload your figure files to the [Preflight Analysis and Conversion Engine](http://pace.apexcovantage.com/) (PACE) digital diagnostic tool.

***Our response:*** *All the figure files have been checked and they meet PLOS requirements. We have also re-referenced the figures in the main text to conform to PLOS Genetics guidelines.*

**Comments to the Authors:**

**Reviewer #1:**

1. The manuscript by Adam Wells and colleagues provides a step up in knowledge concerning the roles of Rab1, Rab6 and Rab11 in DCG formation in the secondary cells of the Drosophila accessory gland. The most significant advancement relates to the development of a live imaging technique that allowed the visualization of these cells and the follow-up of vesicle compartments and their association with endogenously-tagged Rab proteins during biogenesis and maturation.

***Our response:*** *We agree that the study is a step up from previous work. We also agree that the development of real-time imaging of secretory compartments in secondary cells is a significant advance. However, we think our findings are of additional significance, because the size of these compartments and genetic tools available in Drosophila have allowed us to visualise the biogenesis of ILVs and DCGs at a level of resolution that we believe is not currently possible in other systems. This has been instrumental in informing our interpretation of the subcellular knockdown phenotypes that we observe.*

2. However, the Rabs transitions, specifically the Rab6 to Rab11 transition that is claimed in the title of the manuscript as required for DCG formation and that would represent the novelty and most significant contribution of the work is not directly tackled.

***Our response:*** *We think that this is a key aspect and that we have directly tackled the requirement of the Rab6 to Rab11 transition (‘the process of changing from one state to another’) for DCG biogenesis, most notably through the knockdown of Rab11 in adult secondary cells (SCs). This provided strong evidence that Rab11 is required for DCG assembly and that in its absence, large non-acidic compartments are formed, but they do not generate DCGs (Fig 7C, D). These compartments are labelled by Rab6 (Fig 8F, G). We note the points made by this reviewer below concerning other approaches to assess the interactions between Rab6 and Rab11, and have addressed these in the revised manuscript and in this document.*

3. It is important to mention that the presence of these three Rabs on the membrane of these vesicular compartments had been previously reported, as well as its requirement in the development of DCG.

***Our response:*** *Regarding SCs, localisation of Rab11 on large DCG compartments has been previously reported (Corrigan et al., 2014; Redhai et al., 2016, etc). Rab 6 localisation was also reported in Prince et al., 2019, and shown to co-localise with Rab11 on some compartments, but DCGs were not analysed in this study. In fact, because the Prince et al. analysis was primarily undertaken with fixed tissue and most of the experiments used fluorescently labelled Rabs that were marked by YFP, it was not straightforward to determine which compartments were co-labelled with different Rabs in the absence of other morphological features, such as DCGs, which typically need to be fluorescently labelled to be visualised in fixed glands. A good example from that study is the Rab19-positive large compartments, which were proposed (through indirect knockdown evidence) to be linked to Rab7 trafficking in Prince et al., 2019, but which we have clearly shown are a subset of more mature DCG-containing, Rab11-compartments. On the website associated with the publication by Prince et al., 2019, Rab1 and Rab2 were reported to be located in a central apical mass, but this was not further characterised, so again our analysis has advanced our understanding.*

*A critical advance in our current studies, facilitated by real-time imaging, is to demonstrate that the Rab6-compartments, which are not Rab11-positive, transition to a Rab11 identity (Fig 3). The compartments do not merely deliver their contents to Rab11-compartments. In our study, the Rab1 to Rab6 transition is also observed in SCs for the first time (Fig 2), though it has previously been reported in yeast (Thomas et al., 2021). The ability to follow compartments in real-time as they change from one identity to another, highlighted by this reviewer in point 1, has allowed us to characterise the dynamic events taking place, which is very challenging in any system where only fixed tissue can be used or high-resolution real-time imaging is not applicable.*

*Regarding the requirement for the different Rabs in DCG biogenesis, Rab6 knockdown was reported to block the formation of large compartments labelled by Rab6, 7, 11 and 19 (Prince et al., 2019). However, we have shown that some large non-acidic compartments are still present in these knockdown cells, but DCGs are absent. One critical distinction between our experiments and those undertaken by Prince et al., 2019, is that they knocked down genes throughout the development of SCs from pupal stages onwards, whereas we have only knocked down genes after eclosion, using the GAL80 temperature-controlled system. This allows us to focus our analysis on the period of time when SCs start to make large secretory compartments (the time course of this process is described in Prince et al., 2019) and we therefore specifically analyse the maturation processes that we wish to study. This alternative focus and its rationale, which we have now emphasised more in the Results section (line 314 onwards), is also considered in point 1, Rev. #2, in the context of demonstrating the specific requirement of Rab6 and Rab11 in the formation of DCG compartments.*

*In respect to the roles of Rabs in DCG biogenesis, Prince et al., 2019 did not score DCGs, since in fixed tissue, as mentioned above, this typically requires DCG-specific markers (Redhai et al., 2016). This is important, because we have shown, for example with many ESCRT knockdowns, that small non-acidic compartments can still make DCGs (Marie et al., 2023). Hence, showing a reduction in compartment size following Rab6 knockdown in Prince et al., 2019 was not sufficient to conclude that DCG biogenesis is blocked. We do not think that the role of Rab11 in SC DCG biogenesis has been tested previously, nor has it been considered in the context of the Rab6 to Rab11 transition that we have identified.*

*Our focus in this manuscript is primarily on DCGs, but we should also point out that our work has revealed the trafficking of Rab6 on to ILVs for the first time (Figs 1I and 1M; S1B and S1H Fig) and shown the requirement for Rab11 to allow this Rab6 trafficking to ILVs to take place (Fig 8M), consequently providing further evidence for a link between processes controlling ILV biogenesis and DCG formation, discussed previously in Marie et al., 2023. We therefore think that our manuscript is a substantial advance on previously published findings concerning Rab localisation and function in SCs.*

4. These are descriptions of the live imaging observations and **no mechanistic regulation of the process is provided.**

***Our response:*** *We agree that the real-time imaging that we present is descriptive, but as alluded to by us above, highlighted by Rev. #1 in point 1, and discussed further below, it is the critical foundation upon which our subsequent knockdown analysis is based and interpreted. The knockdown analysis provides mechanistic insights, particularly the analysis of Rab6 and Rab11 functions in the generation of DCG compartments and DCG precursor compartments. However, this would be very difficult to interpret fully without the foundation of our live cell imaging and its demonstration that Rab6-only compartments directly mature into Rab11-positive DCG compartments.*

5. Also, they detect that these Rab6-positive DCG-negative compartments continue its maturation process by: incorporation of Rab11; internalization of Rab6 and ILV formation, resulting in a compartment with Rab11 externally, Rab6 in ILV and a DCG. **Does the Rab6-Rab11 transition require inactivation of Rab6 or its dissociation from the maturing DCG-containing compartment? Is all Rab6 incorporated into ILV? What happens if Rab6CA is expressed?**

***Our response:*** *These are all interesting questions. Data that addresses some of them were presented in the original manuscript, and we have now flagged these findings more clearly in the Results and Discussion sections (lines 207-210 and 683-685), but we have also followed the helpful suggestions of this reviewer and further expanded the experimental analysis (see below).*

*Regarding our strategy in the original manuscript, we have favoured the use of Rab knockdowns instead of overexpressed mutant Rabs to test function for three major reasons. First, we find that overexpression of some wild type Rabs produces significant effects on SC compartment organisation, eg. tubulin-YFP-Rab11 reduces trafficking to lysosomes in SCs (Corrigan et al., 2014). This potentially affects our ability to interpret experiments where YFP-labelled mutant Rabs are overexpressed. Second, publicly available mutant Rabs are YFP-labelled, preventing us from using the GFP- and YFP-marked proteins that we have employed in our study to assess the effects of these mutants on DCGs and other Rabs. Third, in our hands, the effects of mutant Rabs can be very variable in vivo. Overexpressed dominant-negative Rab mutants sometimes do not affect membrane-binding of their wild type equivalents, perhaps because they are not expressed at sufficiently high levels. And if overexpressed constitutively active Rab mutants do not produce a phenotype, it can be difficult to ascertain whether they are behaving as CA mutants in the specific cellular context under test.*

*Regarding our knockdown experiments, once we had performed them, it became clear that although some of the RNAis were probably only achieving a partial knockdown, for other RNAis that had previously been employed in other studies, there was a very strong effect on DCG compartment biogenesis (see also point 1, Rev #2, where these latter published RNAi studies are listed; this issue is also discussed in points 6 and 9, Rev #1 below). We could only properly interpret any knockdown phenotypes using the YFP-Rab11 and GFP-GPI lines (Figs 7 and 8), which we could not employ with the mutant Rabs highlighted by this reviewer.*

*In terms of the specific questions raised by the reviewer:*

Does the Rab6-Rab11 transition require inactivation of Rab6 or its dissociation from the maturing DCG-containing compartment? : *The data in the original manuscript reveal that Rab6 remains attached to DCG compartments as they transition to Rab11-positive compartments and is ultimately present on about 40% of DCG compartments (Fig 1L). Individual new DCG compartments are generated from the trans-Golgi network every 4-6 hours (Redhai et al., 2016), so this means some of the DCG compartments are likely to have remained associated with Rab6 for as much as 24 hours after the initial binding of Rab11 to their surface. It seems likely that this Rab6 is still active, but we cannot provide conclusive evidence for this. Unfortunately, the mutant Rab6 experiments that we have now undertaken shed only limited light on this issue (see below). We have commented on this specific point in lines 683-685 of the revised manuscript.*

Is all Rab6 incorporated into ILVs? : *The data we presented in the original manuscript indicated that most of the Rab6 on DCG compartments is at the limiting membrane and only a small proportion is incorporated into ILVs. Much of this Rab6 internalisation appears to occur in the first few hours of the Rab6 to Rab11 transition. We have commented on this point on lines 207-210 of the revised manuscript.*

*We have now performed experiments with mutant Rabs, as suggested by this reviewer (see also point 11). A few of the findings are informative, while several others are difficult to interpret for the reasons given above.*

*For wild type Rab6 overexpression, the DCG compartments appear normal and some YFP-Rab6 is trafficked into ILVs. Rab6-DN induces a very strong multi-compartment non-DCG phenotype. However, overexpression of this mutant Rab6 leads to unusual trafficking of the molecule, with much of it imported into the two nuclei of SCs. It does not appear to specifically localise on membranes of the numerous compartments produced. The compartment phenotype broadly resembles the Rab6-RNAi #1 knockdown phenotype, where multiple small compartments that lack DCGs are observed (Fig 7B). Surprisingly, the phenotype becomes more variable when Rab6-CA is co-expressed with wild type CFP-Rab6 produced from the endogenous Rab6 locus: a few cells have the strong multi-compartment phenotype, but many others appear much more normal and Rab6-DN becomes associated with Rab6-positive compartment membranes. It is unclear whether this means that this Rab6 mutant, which should lack GTP-binding activity, can bind membranes in its GDP-bound state or whether it does not completely block GTP binding. Finally, Rab6-CA does not appear to induce a phenotype and it seems to mark similar numbers of DCG compartments to wild type Rab6 (confirmed by CFP-Rab6), suggesting that in the context of adult SCs, Rab6 can be active on Rab11-positive DCG compartments. However, even the constitutively active form can detach from these compartments, as they mature. These new data are presented in a new Supplementary Figure, S8 Fig, and discussed in the Results and Discussion sections (lines 463-505 and 670-675).*

6. Then they analyze the requirement of Arf1 and components of the Ap-1 complex in this process,
regulators previously characterized in mammalian cells as important for DCG formation. Important to say is that these proteins are known regulators secretory vesicle emergence from TGN. Therefore, they are expected to be required in general for DCG compartment biogenesis and specifically for DCG formation. Therefore, in this reviewer opinion this particular point adds on to previously described and extensively characterized role of Arf1 and AP-1 on TGN-derived vesicle biogenesis.

***Our response:*** *We agree that the primary objective of our analysis, when we initiated experiments with Arf1 knockdown and knockdown of AP-1 subunits, which was highlighted in the subtitle of this results section (‘DCG biogenesis in SCs is regulated by evolutionarily conserved mechanisms’), was to confirm that DCG biogenesis in SCs is controlled by some of the same general regulators as in mammalian cells. This is an important point because the DCG compartments in SCs are thousands of times larger in volume than those in mammalian cells and it might easily be argued that they are therefore not equivalent structures and therefore may be under very different regulation.*

*Since* *we have multiple live markers relevant to DCG compartment maturation, eg. CFP-Rab6, YFP-Rab11 and GFP-GPI for the DCG itself, our characterisation allowed us to show critical functions and potential differences in the phenotypes for Arf1 and AP-1 knockdown, which we discussed in the original manuscript both in the Results and Discussion sections. However, the text has been revised and hopefully clarified based on the helpful comments from Revs #1 and #2 (eg. lines 346, 369, 399, 639, etc). Most notably, we have placed somewhat more weight on the findings obtained with the strongest RNAis for these regulators, Arf1-RNA1 #1 and AP-1γ-RNAi, which have both been used in previous studies; in combination with the GFP-GPI DCG marker, they produce SCs that almost exclusively have no DCGs (Figs 4B, 4C and 4F). However, we show that AP-1γ knockdown cells retain the capability to form multiple large Rab6-positive compartments using the CFP-Rab6 marker (Fig 6H). Often, they also have a central cluster of small Rab6-positive compartments in the location of the trans-Golgi (Fig 6C). By contrast, for both Arf1 knockdowns, multiple large non-acidic compartments are formed that are not marked by Rab6 (Fig 6B and 6H; S6D Fig) or Rab11 (Figs 5B and 5F, S5D Fig). The central clusters of small Rab6-positive compartments seen in AP-1γ knockdown cells are also not observed. We could only easily assess Rab1 identity for Arf1-RNAi #2 knockdowns, but in this background, we found that at least some of the large Rab6-negative compartments are labelled by Rab1 (Fig 6G; see also point 7 below).*

*Overall, we agree with this reviewer that the Arf1/AP-1 aspect of our work does not represent a major step forward in our understanding of these two regulators. However, it does provide a clearer link between these regulators and the Rab6/Rab11 trafficking molecules that we show also regulate compartment maturation, and it suggests somewhat different roles for these regulators.*

7. Similarly, the reduction in Rab11- and Rab6-labelled compartments upon Arf1 and AP-1 downregulation is expected since no Golgi-derived vesicles are generated when the function of these crucial factors is compromised. Supporting this is the observation made by the authors upon expression of the same RNAis in a genetic background that expresses Rab1-YFP. They detect that Rab1 compartment is enlarged consistent with cargo accumulation at TGN upon Arf1 and AP1 silencing. Again, these are not novel functions for these proteins/complexes.

***Our response:*** *We agree with the reviewer that inhibiting Arf1 and AP-1 expression would be expected to suppress release of immature DCG compartments from the TGN. As indicated in point 6 above, we have now more clearly highlighted the phenotypes seen with the strongest Arf1 and AP-1 RNAi lines, Arf1-RNAi #1 and* *AP-1γ-RNAi, which have both been previously used to study these molecules in flies (Wang et al., 2017; Peterson and Krasnow, 2015). However, we believe that our data actually suggest differences in the effects of AP-1 knockdown versus Arf1 knockdown. As the reviewer points out, for Arf1 knockdown, some of the large non-acidic, non-DCG compartments formed are marked by YFP-Rab1 (Fig 6G), and not Rab6 (Figs 6B and 6H and S6D Fig; see text, line 387 onwards). However, for the AP-1γ knockdown, these large compartments are typically marked by Rab6. As mentioned in point 6, there is also often an accumulation of small Rab6-positive compartments around the central region occupied by the Golgi in SCs, suggesting that compartments typically progress past the Rab1 to Rab6 transition in the maturation process (Fig 6C and 6H). We have adjusted the text slightly to clarify this point both in the Results and Discussion sections. We believe our findings may provide some additional insight into the functions of these molecules at different stages of DCG biogenesis, but we appreciate there are limitations to how far we can take our conclusions (line 390 onwards and line 639 onwards).*

8. In this point the authors draw a conclusion with which this reviewer disagrees: “The existence of these large compartments marked by Rab1 also suggests that Rab6 is not required for the fusion events between compartments formed in the trans-Golgi and compartment expansion to
occur”. The authors interpret that Rab1-positive large compartments are a consequence of Rab6-
independent homotypic fusion whereas this reviewer tends to think that they are an enlarged Golgi
complex that results from accumulation of material that is unable to emanate inside Golgi-derived
vesicles. **The authors should provide clear evidence to distinguish between these two possibilities.**

***Our response:*** *This is an excellent point, thank you. As we reported in the original manuscript, in normal SCs, we only see the fusion events sporadically and these events only provide part of the explanation for the growth of Rab1/Rab6-positive compartments, which, as our movies show, also appear to enlarge in the absence of fusion. For this reason, we think it would be very challenging to convincingly demonstrate that compartment fusion is either entirely dependent or independent of Rab6 in an Arf1 knockdown background. To attempt this, we would need to generate new lines of flies expressing YFP-Rab1 and CFP-Rab6 markers that also allow Arf1 knockdown, which we have found problematic. We would then need to try to image SCs at 6 days in this genetic background, when some Rab1-positive compartments would already be very large, making it difficult to visualise fusion events with other much smaller Rab1-positive compartments that are likely to reside below the large compartments. Since the Rab6 to Rab11 transition is the focus of our studies, we have decided that we will not focus further on undertaking this challenging experiment. Instead, we mention both the fusion idea and the alternative hypothesis proposed by this reviewer as possible explanations of our data (lines 393-399).*

9. To support the idea that the Rab6 to Rab11 transition is required for DCG biogenesis, the authors should perform experiments to test this more specifically, and not by affecting TGN-derived vesicle biogenesis all together.

***Our response:*** *We have commented on the additional experiments that the reviewer has suggested with mutant Rabs, both above (point 5) and below (point 11), and have undertaken these experiments. However, our view is that out Rab11 knockdown experiments provide good evidence that Rab11 is required for DCG formation. Using the stronger Rab11 knockdown line (RNAi #1), almost all SCs have no DCG compartments in the GFP-GPI marker background (Figs 7C and 7D). Since our live cell imaging data show a very strong correlation between the initiation of ILV/DCG biogenesis and the beginning of the Rab6 to Rab11 transition, we conclude that this transition is likely to be a key process driving these biogenesis events. Rab11 knockdown is very unlikely to be a manipulation that directly affects TGN-derived vesicle biogenesis, and indeed, Rab11-RNAi #1 does not affect the number of large Rab6-positive compartments generated (Fig 8L), which we believe are the precursor compartments formed prior to this Rab6 to Rab11 transition. Of course, as in all cell biology studies, we cannot conclusively exclude the possibility that when one specific trafficking process is blocked, this indirectly affects another secretory or endosomal trafficking event in the cell. However, we believe that the Rab11 knockdown experiment is the most definitive experiment that we could undertake at this time to test the role of the Rab6 to Rab11 transition.*

10. Also, the authors try to define temporal requirements for Arf1 and AP-1 complex depending on the severity of the phenotypes obtained upon their knock-downs. It is advisable to make such comparisons based complete loss of function analysis since the different phenotypes obtained could be dew to different degrees of mRNA interference and not real functional differences. **Additionally, the authors could perform a phenotypic characterization at different levels of RNAi expression (by rising flies at temperatures below 29C).**

***Our response:*** *We agree that it would be optimal to complement our studies with complete loss-of-function analysis, but these experiments are challenging in the SC system for two reasons. First, it is difficult to make clones of mutant cells that consistently include homozygous mutant SCs, and so studies of adult accessory glands from our lab and others have typically focused on knockdown. Second, and more important, we think it is critical to assess the effect of gene manipulation in adult SCs, after these cells have differentiated during pupation. This requires the temperature-controlled GAL4/GAL80 system to block RNAi expression in pupae. This ensures that we only look at the effects of knockdowns as compartments are being formed in adulthood, as opposed to while the cells are initially differentiating, a period during which they do not form any large non-acidic compartments.*

*This temperature-regulated system also restricts our ability to modulate temperature to alter the levels of RNAi expression. There is no RNAi expression at 18 or 25 degrees in our experiments because GAL80 completely inhibits GAL4 activity at these temperatures. As discussed in point 5, Rev #1 and point 1, Rev #2, we have tried to ensure that the knockdown phenotypes are as strong and reliable as possible by using at least one RNAi for each gene or protein complex that has appeared to give a strong loss-of-function phenotype in other publications. In all cases, this RNAi induced almost complete loss of all DCGs in SCs expressing the GFP-GPI marker, while the second RNAi that we used was found to induce a weaker phenotype, but one that is consistent with the other RNAi knockdown. In some instances, the weaker phenotype gives some clues about the defects involved (see also points 3 and 4, Rev. #2). Given the limitations of the cell model we are using and the specific questions we seek to address, we believe that this is the most appropriate way to study the function of these genes. However, as discussed in points 5 and 11, we have also assessed the effects of dominant-negative and constitutively active Rab6 and Rab11 expressed only in adult SCs, which in a subset of cases, provided further support for our findings.*

11. Later on the authors test weather Rab6 and /or Rab11 are required for DCG biogenesis, and find that both of the do. However the effect is not complete, with some DCG still forming. These could be due to remnant levels of wither Rab6 or Rab11 proteins, supported by the fact that Rab6-YFP and Rab11-YFP are still detectable under silencing conditions of the respective mRNAis. Or, more interestingly it could be due to the existence of more than one pathway controlling DCG biogenesis. **These two possibilities should be evaluated. The effect of Rabs dominant negative isoforms could be tested.**

***Our response:*** *The reviewer has raised some interesting points, which have led to us undertake the suggested experiments with dominant-negative Rab mutants. Unfortunately, only the Rab6 dominant-negative mutant produced a strong effect, which potentially supports the hypothesis that Rab6 is essential for DCG biogenesis. However, the mutant protein does not consistently appear to behave as expected in terms of its localisation and effects on wild type Rab6 (S8 Fig), perhaps because of variable expression levels. This mutant Rab6, which is predicted to be impaired in GTP binding, has not been extensively characterised in published work, and so it is difficult to fully interpret this result. For the Rab11 mutants (S9 Fig), the constitutively active Rab11 and dominant-negative Rab11 seem only to be expressed at very low levels in SCs. The dominant-negative Rab11 appears to be excluded from the limiting membrane of large compartments as expected, but it does not interfere with binding of endogenous Rab11, except for perhaps producing a subtle phenotype involving small Rab11 compartments that interact with the large DCG compartments. Therefore, neither of these mutant proteins provides a good tool to study Rab11 function in SCs and neither, in fact, affects the gross morphology of these cells.*

*The points made by the reviewer also indicate that we should have discussed the phenotypes that we observe with different RNAis more clearly in the original manuscript (see also point 5 above). As we discussed in the original manuscript, we observed the strongest phenotypes when RNAis were co-expressed with GFP-GPI. Importantly, for the Arf1, AP-1, Rab6 and Rab11 RNAis producing the strongest phenotypes (see also point1, Rev. #2), almost all SCs characterised had no DCGs (Figs 4B, C and F, Figs 7B, C and D). We therefore believe that we have provided strong evidence that these Rabs are required for DCG biogenesis and that it is unlikely that other parallel pathways are involved. We find that these knockdowns have less strong effects in CFP-Rab6 and YFP-Rab11 backgrounds for reasons we do not understand. However, it is not uncommon for knockdowns to show some variability in their effects in different genetic backgrounds and with different markers. It is notable, however, that SCs with no DCGs almost invariably represent the most common phenotype, even in weaker knockdown backgrounds (Figs 5G, 6I, 8G and 8H), consistent with our proposal that these genes are required for DCG biogenesis. We have now clarified these points more directly in the relevant Results sections and the Discussion of the manuscript (lines 369-382, 451-457, 624-633, 668-670).*

**Reviewer #2:**

The images and associated quantification are high quality and convincingly show the sequence of Rab protein occupancy on the dense core vesicle membranes.

***Our response:*** *This reviewer remarks on the high quality of the imaging and how it convincingly shows the changes in Rab occupancy, including the Rab6 to Rab11 transition.*

Major concerns:
1. No validation of RNAi knockdown efficiency, either by qPCR or by western blot/imaging, is presented in the manuscript. This leads to a perceived lack of experimental rigor and calls into question the validity of the presented RNAi results.

***Our response:*** *This comment relates to an experimental limitation with the system that we use. Secondary cells make up approximately 2.5% of all the epithelial cells in the accessory gland and there are also muscle cells surrounding the epithelium. None of the genes that we are studying are expressed exclusively in SCs, so we cannot assess the level of knockdown by qRT-PCR or Western blotting of dissected tissue. Furthermore, in our hands, there are no antibodies available that produce sufficiently high-quality immunostaining that they could be even semi-quantitative, especially since the stainings will be highly localised within the cell, their distributions will change following knockdown, and as we have seen for the Rab knockdowns, the knockdown level will vary from cell to cell. Because of this limitation, our publications on secondary cells have relied on RNAi knockdown experiments, which in most cases, we have not been able to quantify, eg. Leiblich et al., 2012, Corrigan et al., 2014, Redhai et al., 2016, Leiblich et al., 2019, Fan et al., 2020, Dar et al., 2021, Marie et al., 2023.*

*To maximise the experimental rigour of our approach, we have two major strategies. First, we try to employ RNAis that have been used previously and produce strong levels of knockdown and robust specific phenotypes. For this manuscript, these RNAis are: Arf1 (Wang et al., 2017; both RNAis employed give strong phenotypes with RNAi #1 (v23082) being primarily used in their study and displaying phenotypes similar to dominant-negative Arf1), AP-1γ (Peterson and Krasnow, 2015; gives strong blockade of trafficking from recycling endosomes in tracheal cells), Rab6 (Ayala et al., 2018; RNAi #2 produces knockdown phenotype similar to complete loss-of-function clones, and in this case, RNAi #1 [Zhou et al., 2023] induces somewhat stronger phenotypes) and Rab11 (Ma and Brill, 2021; both RNAis used in our study were employed in this paper with RNAi #1 appearing to give the stronger phenotype). Second, we have tried to circumvent the potential problem of off-target effects by using two independent RNAis for Arf1, Rab6 and Rab11, and RNAis for three different AP-1 subunits. These knockdowns provide strong evidence that all these molecules are involved in SC DCG biogenesis, particularly clearly seen in the GFP-GPI background. In this background, the most common phenotype induced by even the less potent Arf1-RNAi #2, AP-1µRNAis and Rab6-RNAi #2 is a SC with no DCGs. The variation in the severity of the phenotypes observed almost certainly reflects different levels of knockdown, which can be seen even in SCs in which a gene is knocked down by the same RNAi; this biological variation is represented by the scattering of data points in each bar chart.*

*We should have more clearly stated the issues concerning our experimental approach at the start of our knockdown analysis and have now included an explanation in the text (lines 314-333). However, for what appear to be the strongest knockdowns of Arf1, AP-1, Rab6 and Rab11, in the GFP-GPI genetic background, there are essentially no DCGs present in any SC (Figs 4F and 7D). We should have also highlighted these strong phenotypes more clearly in our analysis of the data, because, irrespective of the level of knockdown (which is likely to be high in these cases), it demonstrates a central role of these genes in DCG biogenesis. We have now flagged this more clearly in the text together with the fact that all of the RNAis have previously been used in published studies to knock down their respective genes (lines 327-333, 369-372, 451-453, 631-633, 668-670).*

2. Figs 5F and 6H: How do the authors explain the differing findings with the two Arf1-RNAi lines?

***Our response:*** *As also discussed in points 6 and 7, Rev #1, Arf1-RNAi #1, which has been used extensively in a previous publication (Wang et al., 2017), seems to produce stronger phenotypes than Arf1-RNAi #2. Virtually all Arf1-RNAi #1 knockdown cells contain no DCGs in the GFP-GPI marker background, where we consistently see the strongest phenotypes (Figs 4B and 4F), and most (in many cases all) of the large non-acidic compartments that are produced are not labelled by Rab6 (Fig S5D). We believe that most of these compartments are labelled with Rab1, but we found it difficult to generate the recombinant flies required to produce males containing YFP-Rab1, CFP-Rab6, dsx-GAL4, GAL80 and Arf1-RNAi #1 to show this. However, based on our analysis of Arf1-RNAi #1 knockdown cells, the first step in DCG biogenesis that is blocked by Arf1 knockdown is likely to be the Rab1 to Rab6 transition.*

*Arf1-RNAi #2 seems to produce a weaker knockdown and this leads to some compartments in most (but not all) cells passing through the Rab1 to Rab6 transition. These compartments then appear to be able to mature past the Rab6 to Rab11 transition, hence the higher number of Rab11-positive compartments in this knockdown (Fig 5F), most of which form a DCG (Fig 5G). We therefore conclude that the weaker knockdown with Arf1-RNAi #2 provides sufficient Arf1 in a subset of cells for some compartments to progress through the Rab1 to Rab6 transition, and that if these compartments pass through that transition, they are often then able to form DCGs. However, it is important to note that for Arf1-RNAi #2, the most common DCG phenotype in the GFP-GPI background is absence of all DCGs (Fig 4F), which is consistent with the phenotype seen with the stronger Arf1-RNAi. Furthermore, we show that these knockdown cells contain large non-acidic compartments that are labelled with YFP-Rab1 (Fig 6G), which are not observed in control cells (Fig 1G), again consistent with the idea that Arf1 is involved in the Rab1 to Rab6 transition. We have now discussed this point in more detail as we consider the phenotypes in the Results and Discussion sections of the manuscript (lines 336-337, 372-373, 384-399, 658-665).*

3. “The AP-1γ knockdown, in which, unlike other knockdowns, the average number of Rab6-positive compartments was not reduced compared to controls (Fig 6H), was particularly notable. The number of Rab11-positive compartments was strongly reduced (Fig 5F), and, in most cells, none of the compartments contained DCGs (Fig 6I). This is consistent with AP-1 playing a key role in the Rab6 to Rab11 transition, and the hypothesis that this transition is required for DCG biogenesis.”
-- This argument by the authors does not make sense, because the three AP-1 subunits examined all form a single complex. Thus, knockdowns of all three should show similar phenotypes, unless there is a substantial difference in knockdown efficiency between the three RNAi constructs. How do the authors explain the different phenotypes observed in AP-1gamma knockdown vs. mu and sigma?

***Our response:*** *We agree with this reviewer that if the functional impact of knockdown was equivalent for all the AP-1 subunits, we would see the same phenotypes. Although all three knockdowns significantly reduce the number of DCG compartments formed in the GFP-GPI background (Fig 4), there clearly is a substantial difference in the impact of these different knockdowns on DCG compartment maturation and Rab compartment identity. This could be explained by different levels of knockdown or unequal levels of normal expression for each subunit; it might also reflect an off-target effect for the stronger AP-1γ knockdown, although the knockdown line employed has previously been used in other studies to specifically block the secretory pathway, eg. Peterson and Krasnow, 2015, and the phenotypes in SCs share some similarities with strong Arf1 knockdowns. Knockdown of AP-1μ and AP1-σ consistently gives weaker phenotypes than for AP-1γ with the GFP-GPI, Rab11 and Rab6 markers. Our interpretation of the data obtained with Rab6 and Rab11 markers is that on average, more large non-acidic compartments are able to mature from Rab6 to Rab11 identity and form DCGs for the former two knockdowns (Fig 4F and 5G). However, for the AP-1μ knockdown, it is important to emphasise that in the GFP-GPI background, the most common DCG phenotype is absence of all DCGs (Fig 4F), consistent with the idea that it is behaving like a weaker version of the AP-1γ knockdown in the more sensitised GFP-GPI genetic background. We have now considered these points in more detail in the Results and Discussion sections (lines 346-357, 624-630).*

4. “Our data suggest that Arf1 acts earlier in the process than AP-1, since unlike AP-1, it is required to recruit Rab6 to large compartments, as well as for the later conversion to Rab11-positive compartments. By contrast, AP-1 is primarily involved in the Rab6 to Rab11 transition and in subsequent maturation events that induce DCG formation.”
-- It is difficult to understand how the authors justify these conclusions, given that the phenotypes presented for Arf1 and for AP-1 knockdown are similar… knockdown of Arf1 and AP-1 all reduce the number of Rab11-containing compartments (Fig 5F) and the number of Rab6-containing compartments (Fig 6H, with the exception of AP-1gamma; see the point above). If the authors wish to make this statement, additional experiments will be necessary to justify the stated sequential roles of each protein. Alternatively, the authors could change their conclusions to accurately reflect the shown data.

*Our response: We are grateful for these helpful comments from the reviewer. One additional result not mentioned above, which suggests that Arf1 and AP-1 may not be initially acting at the same point in the DCG biogenesis process relates to the labelling of large non-acidic compartments in different genetic backgrounds. We found that large non-acidic compartments in AP-1 knockdown cells were labelled either by Rab6 or by Rab11, but this was not the case for many of these compartments in Arf1 knockdowns (S6D Fig; lines 387-414, which we have now elaborated on to emphasise this point). In the latter case, we present results showing that some of these large compartments are labelled by YFP-Rab1 (Fig 6G), which is never seen in control cells (Fig 1G). We therefore do have evidence that Arf1 is involved in an early maturation step, the Rab1 to Rab6 transition, in which there is no indication that AP-1 plays a part. In fact, many cells expressing the strong AP-1γ -RNAi have numerous Rab6 compartments, but very few of these form DCGs, and the majority of cells also contain a central cluster of small Rab5-positive compartments in the location of the TGN (Figs 6C, 6H and 6I; lines 408-414). We have reworded the statement highlighted by the reviewer and elaborated on it (line 430 onwards), pointing out the differences that we have observed, which we believe are important to flag, but being somewhat more conservative in the interpretation. We have also highlighted this point again in the Discussion section (lines 624-665), where we consider the roles of these regulators.*

Minor concerns:
5. Full genotypes should be included in the figure legends, since it is unclear if the authors are imaging homozygotes or heterozygotes for tagged proteins. For example, presumably imaging of double-labeled (e.g. CFP-Rab6 with YFP-Rab11) cells is done in heterozygotes; thus, imaging of the single tags should be done in heterozygotes, as well, since 1 vs. 2 copies of the labeled proteins could affect expression levels and thus localization.

***Our response:*** *We agree with the reviewer’s important point concerning heterozygotes versus homozygotes; all of the imaging was performed with heterozygous CFP-Rab6, YFP-Rab11 and UAS-GFP-GPI transgenes, even in the control experiments shown in Figs 1-3. We have now included this information in the figure legends.*

6. Figs 1G-H: How do the authors know Rab1 and Rab2 are labeling Golgi without an independent marker?

***Our response:*** *This is a good point. We did these experiments some years ago, and should have included them. We have now included these data as extra panels in S2 Fig (lines 197-202).*

7. Fig. 1K,L: What criteria were used to define a compartment as having/not having Rab6 or Rab11? The levels of these proteins appear to vary around different compartments… e.g. C’, D’, F’ all have some compartments with bright signal and others faint.

***Our response:*** *This is a helpful point to highlight. In our analysis, it seemed important to count all compartments where there was a clear concentration of Rabs at the limiting membrane relative to cytosolic signal, whether it was strong or relatively weak.* *We therefore scored compartments as positive for the different Rab markers, if the fluorescence intensity at the boundary of the compartment was higher than in the adjacent cytosol. In cases where this was not clear, the transect tool on ImageJ was used to determine whether there was a peak in fluorescence signal at the limiting membrane. The fluorescence signal typically extended all around a compartment, but in the rare examples where this was not the case, the compartment was scored as positive if at least 25% of its perimeter was labelled.* *This excluded the Rab19 labelling of microdomains on some non-acidic compartments. We had explained the basic approach in the Methods section, but have now elaborated on this (lines 831-840).*

8. Fig. 6G: The authors state in the text that the presence of enlarged YFP-Rab1 compartments upon knockdown of Arf1 means that these compartments failed to transition to Rab6-positive compartments. This should be directly shown using live-cell imaging.

***Our response:*** *This is a difficult point for us to address, and this is especially the case, because it has been challenging for us to generate healthy recombinant lines that allow production of males with dsx-GAL4, GAL80, CFP-Rab6, YFP-Rab1 and Arf1-RNAi in them. Point 8, Rev #1 highlights a related issue. In fact, the challenges are also linked to several other points concerning levels of knockdown, discussed above, eg. points 6 and 7, Rev #1; points 2 and 4, Rev. #2, where the approach we use limits our ability to accurately gauge the level of knockdown in our experiments, but also leads to a range of phenotypes being generated in each genetic background that can assist or in some cases hinder interpretation.*

*As we reported, using the stronger Arf1-RNAi #1 knockdown in a GFP-GPI background, we essentially see no DCG formation, but large non-acidic compartments are present (Fig 4F). However, the level of Arf1 knockdown in a CFP-Rab6 background seems to be weaker, because although many of the resulting cells have no DCGs, a few DCGs are formed in some cells, almost invariably within compartments that are smaller than normal (Figs 6B and 6I). If we could generate healthy lines that allowed us to produce YFP-Rab1/CFP-Rab6/Arf1-RNAi #1 males and undertake some time-lapse imaging on these, we might well be able to detect some Rab1 to Rab6 transitions in smaller compartments, but there would probably also be large YFP-Rab1-positive compartments in other cells that might not transition to Rab6 identity. It would probably require a large number of movies to show that these represented different populations of cells with potentially different levels of knockdown.*

*Since the main focus of the manuscript is on the Rab6 to Rab11 transition and not earlier events in DCG biogenesis, we have decided to modify the discussion and interpretation of these data by focusing on two main points that were raised in the original manuscript: (i) the large Rab1-positive compartments observed following Arf1 knockdown are not seen in controls (Figs 6F and 6G), and (ii) several large Rab6-negative, non-DCG, non-acidic compartments are also only observed in Arf1 knockdowns. We use these observations to conclude that a step involved in the maturation process that leads to the formation of Rab6-positive DCG precursor compartments is affected by Arf1 knockdown and not genetic manipulations affecting the AP-1 complex (lines 400-407, 639-646), which we think more accurately reflects our data than our previous interpretation.*

• Reproducibility details should be included in all figure legends.

*Our response: We have explained our general strategy for analysis of knockdowns in the Materials and Methods section and in the first figure in which we show knockdown data (Fig 4). The statistical tests used are given in the Materials and Methods section (see also point 3d, Rev. #3). Ultimately, we analysed three cells per accessory gland from ≥10 males, and because we used live imaging, so we cannot image large numbers of glands at the same time, the data were collected over between three and six different imaging sessions. We have included the n number for each genotype in each legend. The Rab transitions observed during real-time imaging were observed on at least three occasions.*

**Reviewer #3:**

The relative ease in observing large DCGs combined with the genetic tractability of the fly provides a unique opportunity to understand the molecular events involved in DCG biogenesis in an in vivo context.

***Our response:*** *We agree with this reviewer that some aspects of the analysis we have undertaken in SCs are currently uniquely accessible in this system, allowing us to analyse the molecular genetic events underlying DCG biogenesis in ways that are not straightforward to mirror in other cell types and organisms.*

The authors study the biogenesis pathway by observing the localization of different Rab GTPases and find that DCGs, marked by Rab11, originate from small precursor Rab1-labelled compartments. These compartments gradually acquire Rab6 as they mature and eventually acquire Rab11.

Overall, the rationale and experiments are clearly defined, and the model proposed by the authors is well supported by their experiments and is consistent with the literature.

***Our response:*** *We are grateful for this reviewer’s overall assessment of our study and our interpretation of the experiments, and for their careful review of our work, which is considered below.*

Here is a list of minor issues that I feel should be addressed prior to publication:

1. Figures:
a. Most of the panels and graphs are labeled in a font with serifs, which makes the letters and words difficult to read. Please use a bold sans-serif font (Arial, Helvetica, etc.) throughout for clarity, unless the journal insists otherwise.

***Our response:*** *We have changed the font in the figures to bold Arial.*

b. The scale bars throughout are very thin and in some cases barely visible. It would be helpful to thicken the bars to make them easier to see.

***Our response:*** *We have thickened the scale bars in the figures.*

c. Yellow text is not visible on white when printed (Fig. 1E-J, Fig. S1C-E). Black labels could be used instead of yellow, as in Fig. 3. It can be made clear in the legends that the YFP-Rab is shown in yellow in the merged images.

***Our response:*** *Thank you for flagging this. We have made the changes suggested by this reviewer.*

d. The graphs shown in the manuscript are bar charts, not histograms. Figure legends should be corrected to reflect this.

***Our response:*** *Thank you for pointing this out. We have changed this in the figure legends.*

e. The arrows, arrowheads and asterisks in some of the figures are so small that they are nearly impossible to see when printed. This is especially true in Fig. 1, but also to a lesser extent in Fig. 3 and Fig. S1. In addition, the red arrows in Fig. 2E, E’’’ are difficult to see (black might provide more contrast, and the arrows could be larger). This is also true of the red arrows in the panels in Fig. S1D (magenta might provide more contrast, and the arrows could be larger).

***Our response:*** *Thank you. We have made the appropriate changes. For Fig 2E, E’’’, we found that black arrows were also difficult to visualise, so instead, we have outlined the red arrows in black.*

f. In Fig. 2, it would help to label the arrow on top, “Time (minutes)”, for clarity.

***Our response:*** *We have made this change.*

g. Panel labels are missing in Fig. 3 (A-F’’’) and Fig. S2 (A-E’’’).

***Our response:*** *Apologies, thank you for pointing this out. We have now added these – Fig S2 is now S3 Fig.*

2. Movies: It would be helpful to the reader if the movies could be stamped with colored labels to indicate the markers being examined. In addition, the movies should include a scale bar and time stamp.

***Our response:*** *We have included this additional information on the movie files. Thank you.*

3. Methods:
a. Fly stocks: The YFP-Rab lines are not “gene traps” (which suggests a transposable element mediated insertion event). Rather, these are engineered YFP fusions that were generated by homologous recombination at the endogenous loci. The term “gene trap(s)” should be removed from the manuscript and supplementary figure legends.

***Our response:*** *Thank you. We have now corrected this in the different parts of the text that the term has been used, and where required, referred to them as ‘endogenously tagged Rabs’, the term used in the original publication from Suzanne Eaton’s lab.*

b. Fly culture and handling: Six days seems quite long to leave flies at 29°C. Male flies become sterile due to spermatogenesis defects after extended incubation at 29°C. Is it possible the cell biology of the accessory gland is also affected by this extended heat-shock regimen? Are the cells/glands morphologically similar after this treatment to cells/glands from animals not exposed to heat shock?

***Our response:*** *The 29**°C temperature shift is an important aspect of our protocol to ensure that RNAis are not expressed before eclosion and therefore do not disrupt earlier events in SC differentiation. In our hands, we have routinely used this methodology in multiple publications and in some of these, have assessed the fertility of males in different genetic backgrounds, eg. Leiblich et al., 2012, Corrigan et al., 2014, Marie et al., 2023. This also includes studies where we have investigated the cell biology of the main cells, the more abundant epithelial cell type in the gland that makes a large proportion of the seminal fluid (Wainwright et al., 2021). Control males are definitely less fertile than if they were maintained at 19°C or 25°C, but they produce sufficient progeny that we can easily assess a reduction in fertility.*

*It is helpful that the reviewer has made this point, because we have looked at the morphology of SCs at different temperatures when we were first starting these experiments (and with other markers in previous publications). We have now included data relating to cell morphology at 19°C in the manuscript in S1D-G Fig. This shows that there are no significant changes in the number of large compartments marked by Rab6 and Rab11 at 29°C versus* *19°C, nor in the number of these compartments that contain DCGs.*

c. Microscopy: Details about the microscope- the objective and zoom lens combination used to achieve 1000X magnification, laser lines, excitation and emission filters, and camera- are lacking. The authors should also report the acquisition frequency for time-lapse imaging.

***Our response:*** *Thank you. We have included these details.*

d. Statistics: The Kruskal-Wallis test will determine if any condition significantly differs from the control. But the authors must have performed multiple post hoc pairwise testing to identify which specific pairs of conditions are significantly different. As such, the statistics section in materials and methods should include this information. In particular, the authors should clarify whether they have performed some form of correction to the p-values when undertaking multiple pairwise comparisons. This is crucial to control the type I error rate. The differences between control and test conditions are large enough that correcting the p-values for multiple comparisons, if not done already, will likely keep the interpretation the same. However, the tests need to be performed correctly and reported accordingly.

***Our response:*** *Apologies, and thanks for pointing this out. We had undertaken a Dunn’s multiple comparisons post hoc test, and then applied the Bonferroni correction for Type 1 errors. This is now reported in the Materials and Methods.*

4. References: Several references that would provide relevant background or support the authors’ interpretation of their data were not cited (PMID: 21490149, 33866198, 36239631).

***Our response:*** *Thank you for these useful references, which we have included in the Introduction and Discussion sections.*

5. Text: The text contains many complex sentences and is rather wordy. This could be improved by editing for brevity and flow. In addition, moving references to the ends of sentences will avoid choppiness in the text. A partial list of suggested improvements to the writing is included below.

***Our response:*** *Thank you. We have moved references to the end of sentences, where appropriate, made the changes suggested below (except where marked below), and made some additional changes that break down complex sentences in the text to improve the flow.*

Author summary:
p. 3, line 8: suggest replacing “which” with “that”, removing the comma
p. 3, line 15: move comma in “upo,n” to say “upon,”

Introduction:
p. 4, line 3: suggest replacing “secreting” with “producing”
p. 4, line 5: add comma after “neurons”
p. 4, line 6: replace “into” with “in”
p. 6, line 1: cite PMID: 21490149, 33380435
p. 6, line 13: add citation to PMID: 34342349

Results:
p. 8, line 8: delete “in SCs”
p. 10, line 11: suggest replacing “which” with “that”
p. 12, line 6: delete comma after “compartments”
p. 12, line 23: delete “presumably” and change “persists,” to “persist”, removing the comma
p. 15, line 6: change to “Fig. 4B, C, F”
p. 18: line 10: replace “did form” with “formed”
p. 18, line 12: delete “at all. We found that” and start new sentence with “Knockdown”
p. 18, line 13: delete “which lack” and replace with “lacking”
p. 18, line 18: add comm after “knockdowns” and replace “and indicate” with “indicating”
p. 18, line 22: replace “In order to” with “To”
p. 19, line 3: delete “at all in SCs”
p. 19, line 15: replace “where” with “which”

Discussion:
p. 20, line 2: replace “The genetic” with “Genetic”
p. 20, line 15: replace “by” with “at”
p. 21, lines 2-5: suggest moving this sentence earlier in the paragraph to end on a stronger note
p. 22, line 11: delete “knockdown”
p. 22, line15: replace “which” with “that” and remove commas after “materials” and “formation”
Rab19 should be mentioned somewhere in the Introduction, as it comes as a surprise here and in the model figure. – ***Our response:*** *Added in introduction to sentence referring to the Prince et al., 2019 study of SC Rab expression (line 140).*
p. 25, line 1: consider replacing “biogenesis” with “stabilization”
p. 25, line 9: Dar et al., 2021 (PMID: 34795295 is missing from the reference list

Materials and Methods:
p. 26, line 19: replace “which contained” with “containing”
p. 27, line 1: replace “could vary” with “varied”
p. 28, line 3: delete “which”
p. 28, line 10: change “uniform, round and” to “uniform and round, and”
p. 28, line 12: replace “which “contained” with “containing”
p. 28, line 19: add comma to end of sentence
p. 28, line 23: delete “the” after “representing”

6. Figure Legends: The Figure Legends would benefit from editing to remove sentences that restate the Results.

***Our response:*** *We have looked carefully at this issue for the different figure legends, also taking into account the advice provided in the PLOS Genetics guidelines for figure legends: ‘Describe the key messages of a figure: provide a description of the figure that will allow readers to understand it without referring to the text.’ We have therefore retained a number of statements concerning our results in the legends to assist the non-expert reader in interpretation ‘without referring to the text’. We have found in previous publications that non-experts need some guidance in the figure legends to make the images and combined data analysis accessible.*

*The edits below have been made. Thank you for pointing these out.*

p. 39, line 24: define “large compartments” (specify greater than what diameter)
p. 41, line 21: change “compartments” to “compartment” (only one is shown). ***Our response:*** *there are two compartments marked, but we have changed the wording to reflect the point that they are marked by differently coloured arrows.*
p. 41, line 22: change “have to “has”
p. 42, line 10: specify whether the “highlighted compartments” are marked by the blue and yellow arrows
p. 47, line 5: add comma after “Rab19”
p. 47, line 12: add comma after “identity” and delete “and”
p. 47, line 13: change “which” to “and” and “and no” to “or”
p. 47, line 15: delete “their”
p. 47, line16: add comma after “membrane” and replace “which induces the” with “inducing”
p. 47, lines 21-22: delete “Redhai et al. (2016) previously demonstrated that” and start sentence with “BMP signalling”
p. 47, line 24: insert “(Redhai et al., 2016)” after “event”; also change “We additionally show here that AP-1…” to “Additionally, AP-1…”
p. 48, lines 1-2: replace “matured” with “mature” (two instances”
p. 48, line 3: replace “was previously shown” with “is”
p. 48, line 4: replace “what” with “the”, delete “are”, and change “remains” to “remain”
p. 48, line 5: delete “to be” and “compartment”

Fig. S1 legend, lines 5, 9: replace “chains” with “bridge-like structures” for consistency with the Results section
